# The uncertain role of substandard and falsified medicines in the emergence and spread of antimicrobial resistance

**DOI:** 10.1038/s41467-023-41542-w

**Published:** 2023-10-03

**Authors:** Sean Cavany, Stella Nanyonga, Cathrin Hauk, Cherry Lim, Joel Tarning, Benn Sartorius, Christiane Dolecek, Céline Caillet, Paul N. Newton, Ben S. Cooper

**Affiliations:** 1 NDM Centre for Global Health Research, Centre for Tropical Medicine and Global Health, Nuffield Department of Medicine, University of Oxford, Oxford, UK; 2 Medicine Quality Research Group, Centre for Tropical Medicine and Global Health, Nuffield Department of Medicine, University of Oxford, Oxford, UK; 3 Infectious Diseases Data Observatory, Centre for Tropical Medicine and Global Health, Nuffield Department of Medicine, University of Oxford, Oxford, UK; 4 Mahidol Oxford Tropical Medicine Research Unit, Faculty of Tropical Medicine, Mahidol University, Bangkok, Thailand; 5 School of Public Health, Faculty of Medicine, The University of Queensland, St Lucia, Australia

**Keywords:** Infectious diseases, Pharmacodynamics, Population dynamics, Antimicrobial resistance

## Abstract

Approximately 10% of antimicrobials used by humans in low- and middle-income countries are estimated to be substandard or falsified. In addition to their negative impact on morbidity and mortality, they may also be important drivers of antimicrobial resistance. Despite such concerns, our understanding of this relationship remains rudimentary. Substandard and falsified medicines have the potential to either increase or decrease levels of resistance, and here we discuss a range of mechanisms that could drive these changes. Understanding these effects and their relative importance will require an improved understanding of how different drug exposures affect the emergence and spread of resistance and of how the percentage of active pharmaceutical ingredients in substandard and falsified medicines is temporally and spatially distributed.

## Abstract (web summary)

Substandard and falsified medicines are a problem, particularly in low- and middle-income countries, and effects on antimicrobial resistance development aren’t well understood. Here, the authors discuss mechanisms by which they can increase or decrease levels of resistance and the need for improved data collection and analytical approaches.

## Introduction

Substandard and falsified (SF) antimicrobials are a neglected public health problem. Substandard medicines are ‘authorized medical products that fail to meet either their quality standards or their specifications, or both,’ due to within-factory errors or degradation in supply chains^1^. Falsified medicines ‘deliberately/fraudulently misrepresent their identity, composition or source’^1^. Both types of poor quality product can contain too much or not enough active pharmaceutical ingredient (API), no API, an API different to the stated one, and/or fail dissolution testing^1^. Several studies suggest that around 10% of antimicrobials taken by humans may be substandard or falsified in low- and middle-income countries^2–4^. In surveys in parts of Southeast Asia and sub-Saharan Africa, local prevalences >50% have been reported, though those figures should be interpreted with caution^2–4^. Knowing the true extent of this problem is difficult, as sampling is uneven and potentially unrepresentative^5^. This is in part because the majority of surveys use a ‘convenience’ sampling strategy, meaning there is no specific guidance on which locations to sample, potentially leading to biased sampling and some areas not being sampled^4,5^. There have been very few published multicountry studies of antimicrobial quality using a common protocol^6^. It can also be very challenging to sample in remote locations and from unlicensed outlets. SF antimicrobials can lead to worse patient outcomes^2,7–9^, putting an increased burden on already strained healthcare systems and economies. They may also be an important driver of the emergence and spread of antimicrobial resistance (AMR)^10^. AMR is a global public health problem, with resistance to antibiotics causing an estimated 1.3 million deaths in 2019^11^, which is likely to get worse in the decades to come.

Despite the recognition that SF medicines might be an important driver of AMR, there has been little research attempting to understand and quantify the strength of this link. In vitro studies have demonstrated that degraded rifampicin (a first-line tuberculosis medicine) can select for resistance genes in *Escherichia coli* and *Mycobacterium smegmatis*^12^ and that subtherapeutic levels of dihydroartemisinin (a key malaria medicine) can favor resistant strains of *Plasmodium falciparum*^13^. There remain many gaps in our understanding of how SF medicines affect AMR. For instance, how might SF medicines affect the emergence of AMR in human hosts, and how is this affected by the level of API in the medicine and its bioavailability? How much does this affect transmission? What are the population-level impacts of these effects on the prevalence of AMR? In this paper, we first discuss the ways in which SF antimicrobials could impact AMR, and highlight literature relevant to these mechanisms. We end the paper by discussing what types of data and analyses would help us to better understand the relationship between SF medicines and AMR. We focus throughout on the impact on AMR, but it should be re-emphasized that SF antimicrobials will also have other direct and indirect negative effects on morbidity and mortality independent of their effect on resistance. For example, one study found substandard antimalarials were associated with a high treatment failure rate of 28.5% during a malaria epidemic in a refugee camp in Pakistan^9^. Another study found that 3.75% of all under-5 deaths in sub-Saharan Africa could be associated with SF antimalarials, albeit with much uncertainty^14^. Regardless of their effect on AMR, SF antimicrobials remain a severe problem that we must do our utmost to reduce.

## Emergence of resistance

The traditional approach to antibiotic dosing regimens has been based on the principle “Hit hard and hit early”, first formulated by Paul Ehrlich in 1913^15^. This approach was originally formulated as a way to reduce morbidity and mortality due to the infection^16^. It can also be justified, however, as a way to address resistance by ensuring that the dose is high enough to kill even partially resistant microbes^16–19^. Additionally, by shortening the length of treatment the aggressive chemotherapy approach reduces the selection window available for a resistant mutant to emerge^16–18^.

**Fig. 1:**
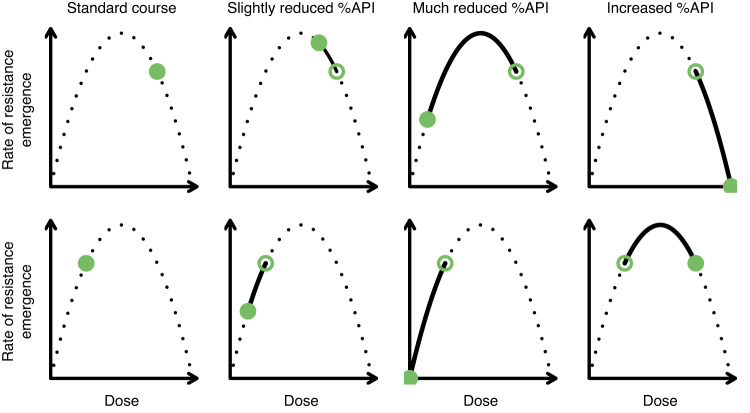
The potential impact of antibiotic dose on the rate of resistance emergence. Based on Kouyos et al.^18^. The rate of resistance emergence is minimized at high doses (when both sensitive and resistant pathogens are eliminated) and low doses (when there is no selective pressure in favor of resistance), and is maximized in between. Closed circles indicate example rates of resistance emergence for different percentages of active pharmaceutical ingredients (API). Open circles represent the rate of resistance emergence with the standard course. The first row indicates a situation where the standard course has a percentage API to the right of the peak in the rate of resistance of emergence, and the second row when the percentage API is to the left of the peak. The difference in the rate of resistance for the closed compared to open circles indicates whether SF medicines will increase or decrease the overall rate of resistance emergence.

However, should a resistant mutant emerge, a higher dose will also give a greater selective advantage to that mutant^18,20^. This trade-off between the density of microbes, which should decline with increasing dose, and the selective advantage of resistance, which should increase with increasing dose, implies that an intermediate dose will lead to a greater rate of emergence of resistance. This has been conceptualized as an inverted-U shape (Fig. 1), and several laboratory studies have found empirical support for this observation^18,21–23^. This observation is related to and expands upon the concepts of minimum inhibitory concentration (MIC), the mutant prevention concentration (MPC), and the mutation selection window (MSW). The MIC is defined as the lowest concentration above which growth of sensitive organisms is inhibited, while the MPC is the lowest concentration above which growth of the least susceptible single-step mutants (i.e. those for which resistance is conferred by a single mutation) is inhibited^24^. The MSW is the difference between the MIC and MPC. The MPC will typically correspond to the right-hand side of the inverted-U, where neither wild-type nor single-step organisms can grow and so the rate of emergence of resistance is zero (Fig. S3). The MIC, however, will typically be slightly greater than the left-hand side of the inverted-U, as there will often be some concentrations above which both sensitive and resistant organisms can grow, but the resistant ones grow more effectively^25^. Hence the inverted-U expands on the MIC, MPC, and MSW concepts by indicating the possibility for sub-MIC emergence of resistance and making it clear that not all concentrations within this expanded MSW will lead to the same rate of emergence of resistance. Given that there is a dose beneath which therapy will not cure the patient, and above which it will be too toxic, the inverted-U implies that in some contexts moderate dosage therapy could be preferred. It should also be noted that the shape of the relationship between dose and the rate of resistance emergence is unlikely to be symmetrical, and is more likely positively skewed^23^. This would occur if either or both of the organism abundance and the selective pressure have saturating relationships with dose, i.e., at high doses the incremental effect of increasing dose is smaller (Fig. S1). As pharmacodynamic effects are typically saturating at high concentrations^26,27^, we might expect that both selective pressure and pathogen abundance would indeed have saturating relationships with dose.

**Fig. 2:**
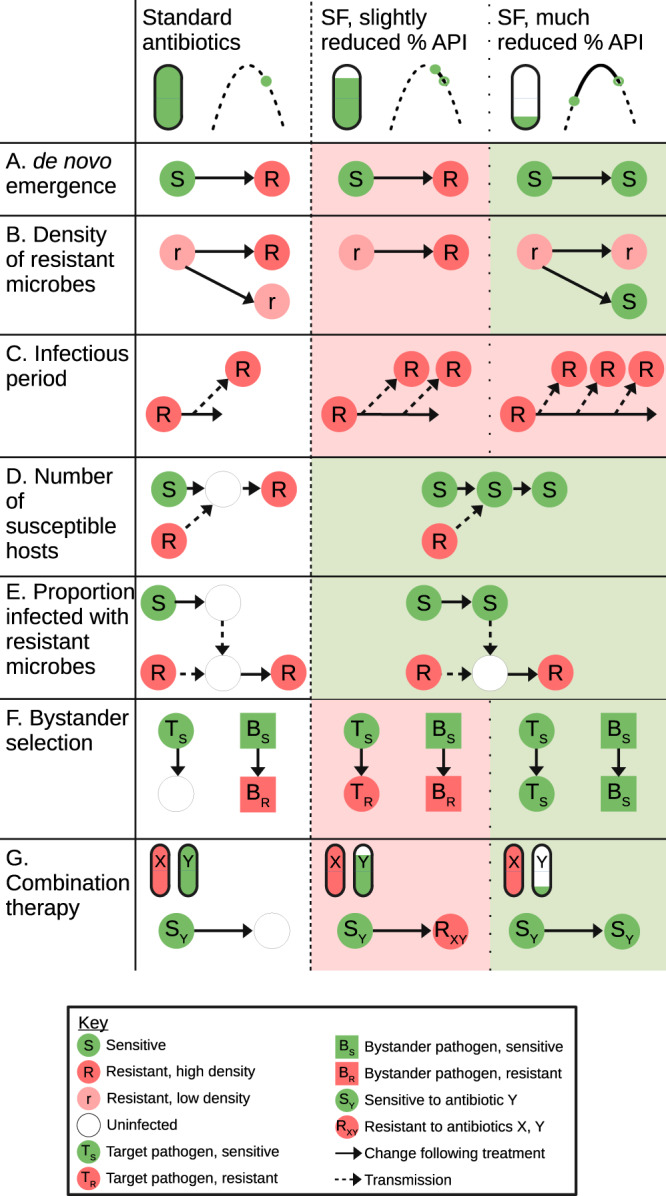
Summary of mechanisms by which substandard and falsified antimicrobials (SF) could affect the emergence of AMR. The first column shows the baseline scenario, and the second and third columns show slightly reduced and much reduced percentages of active pharmaceutical ingredient (API) respectively. For simplicity, we do not show situations with a low baseline percentage API or when API is increased in substandard or falsified antimicrobials. The symbols in the column headers indicate the percentage API and the effect on the rate of resistance emergence (i.e., the inverse of the average time for a resistant pathogen to become established). Solid lines indicate transitions in the same individual, and dashed lines indicate transmission. **A** The effect on the rate of de novo emergence of resistance. **B** The effect on the density of resistant organisms. **C** SF antimicrobials will prolong the infectious periGod, leading to more opportunities for transmission. **D** By reducing the efficacy of treatment, SF antimicrobials could lead to fewer susceptible hosts. **E** Similarly, they could iFncrease transmission from those with sensitive organisms, indirectly reducing transmission from those with resistant organisms. The nature of this effect will also depend on effect A (de novo emergence); if SF medicines increase establishment of resistance, then they could instead increase the proportion of transmission from individuals with resistant infections. **F** Here T refers to the target microbe, and B to a bystander, while the subscript indicates whether they are resistant or sensitive to the antimicrobial. SF antimicrobials could potentially affect bystander selection in the same way they reduce de novo emergence in the target medicine, though the levels of API which lead to the highest rate of resistance emergence will likely differ to that of the target pathogen. **G** Here the subscript refers to the antimicrobial (X or Y) to which they are sensitive (S) or resistant (R). In this example, the pathogen is fully resistant to one of the medicines in the combination, and sensitive to the other.

**Fig. 3:**
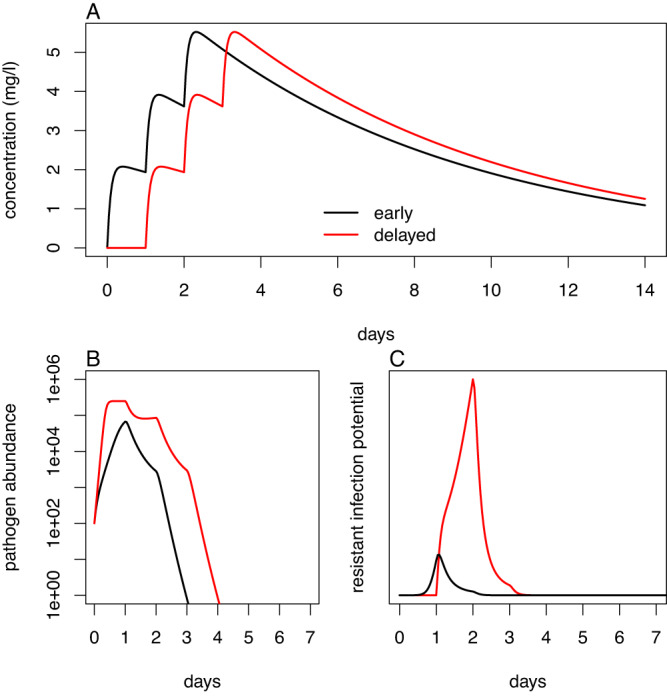
Medicines with no active pharmaceutical ingredient (API) can contribute to the emergence of resistance when followed with a standard treatment regimen. Black lines represent a standard regimen with high-quality antimicrobial taken at time 0, while Red lines represent a standard regimen delayed by 3 days - e.g., because the initial regimen contained no API. A: Example concentration-time curves from a pharmacokinetic model with exponential absorption and exponential elimination. Concentration for a single dose at time 0 is then given by ${{{{{\rm{C}}}}}}\left({{{{{\rm{t}}}}}}\right)=\frac{{{{{{\rm{FD}}}}}}{{{{{{\rm{k}}}}}}}_{{{{{{\rm{a}}}}}}}}{{{{{{\rm{V}}}}}}({{{{{{\rm{k}}}}}}}_{{{{{{\rm{a}}}}}}}-{{{{{\rm{k}}}}}})}\left({{{{{{\rm{e}}}}}}}^{-{{{{{\rm{kt}}}}}}}-{{{{{{\rm{e}}}}}}}^{-{{{{{{\rm{k}}}}}}}_{{{{{{\rm{a}}}}}}}{{{{{\rm{t}}}}}}}\right)$^26^, where F is the bioavailability, here assumed to be 1, V = 931 L is the volume of distribution, D = 2000 mg is the dose, k_a_ = 0.154 hr^−1^ is the absorption rate, and k = 2.00×10^−3 ^hr^−1^ is the elimination rate. The regimen consists of once daily treatment taken for 3 days. B: Example pathogen abundance curves. Pathogen abundance was described by logistic growth and exponential decay (both drug-induced and natural), i.e., $\frac{{{{\rm{dB}}}}}{{{{\rm{dt}}}}}={{{\rm{gB}}}}\left(1-\frac{{{{\rm{B}}}}}{{{{{\rm{B}}}}}_{\max }}\right)-{{{\rm{\alpha }}}}{{{\rm{B}}}}$, where, g = 0.6 hr^−1^ is the growth rate, B_max_ = 1×10^6^ is the carrying capacity, and α is the death rate. Pharmacodynamics are described by a sigmoid relationship between the drug-induced death rate and concentration, i.e., ${{{\rm{\alpha }}}}={{{{\rm{\alpha }}}}}_{0}+\frac{\left({{{\rm{\kappa }}}}-1\right){{{\rm{\alpha }}}}{{{\rm{C}}}}}{{{{\rm{C}}}}+{{{{\rm{C}}}}}_{50}}$, where, α_0_ = 0.3 hr^−1^ is the natural death rate, κ = 5 is the maximum proportional increase in death rate, and C_50_ = 10 mg l^−1^ is the concentration at which the death rate is at half its maximum value. C: Example resistant infection potential (RIP) curves. The area under these curves is proportional to the potential for onward transmission of acquired resistance, and is higher with delayed treatment. The quantity is based on Grenfell et al.^71^, and is given by ${{{\rm{RIP}}}}\left({{{\rm{t}}}}\right)\propto {{{\rm{B}}}}\left({{{\rm{t}}}}\right){\int }_{0}^{{{{\rm{t}}}}}{{{\rm{mB}}}}\left({{{\rm{t}}}}\right){{{\rm{C}}}}\left({{{\rm{t}}}}\right)$, where m is the mutation rate. We assume that the proportion of resistant mutants that become fixed is proportional to C, but the qualitative pattern would also hold provided that this proportion was a monotonic function of C.

The effect of SF antimicrobials on the emergence of resistance will depend on the amount and bioavailability (the degree to which the API is made available at the site of infection) of API in the standard course with high-quality antimicrobial, and how this differs in the SF medicine (note that while we tend to refer to the % API throughout, the actual effect will typically depend on the % API content and the product’s bioavailability). This can be thought of as moving left or right along the inverted-U - if the bioavailable API (closed circles in Fig. 1) in the medicine has a higher rate of resistance emergence than the baseline dose (open circles in Fig. 1), then SF medicines will contribute to the emergence of resistance. In some cases, depending on the mechanism of action, the absorption rate of the API would also be a consideration as a lower absorption rate would reduce peak drug concentrations. SF medicines can also contain an API that is different from the stated one^28^. If the medicine contains slightly less of the stated API than expected (or has reduced bioavailability because it fails to dissolve and absorb properly), then SF medicines could lead to an increased rate of emergence of resistance (Fig. 2A). But if they contain much less API, no API, or an API to which the pathogen is not sensitive, then SF medicines could lead to a lower rate of emergence of resistance - but, of course, the clinical outcomes would likely be much worse. Given that the relationship between rate of emergence and dose is likely positively skewed, it may be that only large or total reductions in API would lead to reduced resistance emergence (Fig. S1). Another important caveat to this is that if someone taking SF medicines later takes a standard course of a high-quality antimicrobial, this could lead to a higher rate of resistance emergence. For instance, hyperparasitaemia is a determinant of the emergence of resistance to antimalarials^17^, due to the high parasite burden increasing the probability for a resistant mutant to arise. If failed treatment with SF antimalarials containing no or little API leads to hyperparasitaemia, which is then treated with a standard course of a high-quality antimalarial, then this could lead to an increased risk of a resistant parasite population being selected for and transmitted (Fig. 3)^7^. Relatedly, SF medicines with too much API may lead to increased toxicity and cause patients to prematurely stop treatment. If this means that the infection is not cleared, then it could lead to an increased risk of transmission of a resistant strain (Fig. S2).

## Transmission of resistant microbes

The key factors that will determine how both substandard and falsified medicines impact on drug-resistance are % API and bioavailability. These factors affect the transmission of resistant microbes via several mechanisms, modifying the mechanisms proposed by Lipsitch & Samore^29^. First, treatment with reduced % API or less bioavailable antimicrobials could affect the density of resistant microbes in an infected individual differently to the standard course with high-quality antimicrobials (Fig. 2B; see also Figure panel D in Lipsitch & Samore^29^). If an individual is infected with a resistant pathogen at a low level, but then receives treatment which gives the resistant organism a competitive advantage or enables it to grow to a higher population size, then this may result in increased density of resistant microbes, which in turn could lead to increased onward transmission. But if the patient received a course of medicines that had little API (or so much that it killed even the resistant microbes), and hence did not give a competitive advantage to resistant microbes, then treatment with SF medicines may result in a lower density of resistance relative to the standard course.

Second, treatment with SF medicines that have a reduced level of API or bioavailability would likely lead to a longer period of infection than the standard course, as pathogens are cleared more slowly, increasing the amount of onward transmission, potentially with resistant organisms if they are present (Fig. 2C). Likewise, treatment failure could lead to chronic infections and hence more onward transmission. For example, Challenger et al. showed that delayed and non-adherent treatment can cause increased transmission of malaria, largely due to increased rates of treatment failure^30^.

Third, relative to the standard treatment course, treatment with SF medicines could reduce susceptibility to infection with a resistant strain (Fig. 2D), and can increase transmission from those infected with a sensitive strain (Fig. 2E). Both of these mechanisms occur as those with sensitive infections recover, and are then susceptible to re-infection with a resistant strain in the former case, and are no longer contributing to transmission thereby creating an opportunity for transmission of a resistant strain instead in the latter case. In both cases SF medicines are likely to reduce these effects by reducing the likelihood of successfully clearing the infection.

Where there is little heterogeneity, the population-level distribution of resistance might be expected to also follow an inverted-U relationship with the average level of API found in medicines. In practice, however, transmission will be moderated by the frequency of AMR in the community and the spatial distribution of SF medicines, and the extent to which these overlap in space and time. In places where AMR is relatively common, this will make the effects described in 2D and 2E relatively more important, as there will be more opportunities for transmission of resistant strains. It is unclear what the impacts of a heterogeneous distribution of SF medicines might be, but if it was such that it led to many patients frequently switching between good quality and SF antibiotics, this could lead to a combination of higher pathogen densities from treatment failure and selection pressure in favor of treatment from the intermittent standard therapy. There are also likely to be lagged effects of substandard and falsified antimicrobials on the future frequency of AMR due to transmission of resistant strains.

## Bystander selection

For many potential pathogens, such as those that often form part of the normal gut flora, most antibiotic exposure that they receive occurs when they are not the target of treatment^31^. This exposure creates a selective pressure in favor of resistant bacteria, known as bystander selection. Failure to account for this effect can lead to mistaken inferences about the effect of interventions to reduce antibiotic use^32^. In addition to resistance in the bystander microbes, this resistance could in some cases be transferred to other microbes via horizontal gene transfer^33^.

In the case of SF antimicrobials, we might expect a similar dynamic to that shown in Fig. 2A, whereby the success of newly emergent resistant strains depends on the level of API in the medicine (Fig. 2F). However, the specific relationship between dose and the rate of emergence of resistance (i.e., the shape of the “inverted-U”) will be different for the bystander pathogen and the target pathogen, and so could result in a situation where the SF medicine reduces resistance emergence for the target pathogen, but increases it for the bystander. We might expect this to be the case when the SF medicines have >100% API, thereby successfully killing all of the target pathogens, but perhaps increasing selection among bystander pathogens. When the SF medicine has little or no API, it will likely reduce the rate of within-host resistance emergence in both target and bystander pathogens compared with use of non-SF medicine. For antibiotics, the extent of the bystander effect will also depend on how broad spectrum the antibiotic is^34^. Unless they have little or no API, SF broad-spectrum antibiotics will have a greater effect on non-target bacteria, and will affect a wider range of bacteria. Bystander effects are further complicated by antibiotic-induced microbiome dysbiosis, that is, when a course of antibiotics reduces the diversity and frequency of commensal bacteria in the gut^35^. If later infection with pathogenic bacteria then occurs, it can lead to more effective establishment and growth of that bacteria due to reduced competition^35^.

## Combination therapy

Antimicrobials are often given in combination, both because such combinations can be synergistic and to guard against the development of resistance^36–39^. For each of the ‘big three’ infectious diseases of malaria, tuberculosis, and HIV/AIDS, the standard regimens are combination therapies: artemisinin-combination therapy for malaria^38^, a four-drug combination of antibiotics for tuberculosis^40^, and a number of regimens for HIV/AIDS^41,42^. As combination therapy often has the intention of making the development of resistance more difficult, consequently the advantages of aggressive therapy increase^18^ and with it the risks of treatment with SF medicines with an insufficient amount of API or poor dissolution features. If one or more of the medicines involved in such combinations are SF, then it could lead to a negation of the synergistic effects or even inadvertent monotherapy. For example, if the pathogen was already resistant to one of the medicines in a two-drug combination, and the second one was substandard or falsified, this could help generate resistance to both medicines, negating the whole combination (Fig. 2G).

The effect of combination therapy is further complicated by the potential for competitive release of resistant organisms by aggressive treatment and how this interplays with the duration of therapy. Peña-Miller et al. showed that synergistic combinations that were more potent than mono-therapies early in the treatment course can later lead to higher bacterial burden than mono-therapy^37^. This occurs when the combination therapy so effectively removes the sensitive population that pre-existing or newly-emerged resistant organisms have no competition and, as a consequence, the resulting overall bacterial burden increases. To avoid this risk, combinations need to also either eliminate resistant pathogens, or enable the immune system to do so^19^. In the context of SF medicines, inadvertent monotherapy or a reduced API in some of the included medicines could on the one hand lead to increased resistance if it results in clearing of sensitive strains but not resistant ones. On the other hand, however, it could lead to reduced resistance in the presence of multi-drug resistant strains, as inadvertent monotherapy may reduce the strength of their competitive release. The specific effect of SF medicines on combination therapy will depend intimately on the disease and combination in question. Although there are currently no direct examples of SF medicines undermining combination therapy, the example of malaria provides an indication of how this could occur (Box 1)^7^, and it has been speculated that SF antimalarials may have been a driver of the emergence of partially artemisinin-resistant *P. falciparum* strains^43^.

Box 1 Substandard artemisinin-based combination therapies and the emergence of antimalarial resistance The standard treatments for Plasmodium falciparum malaria are artemisinin-based combination therapies. These treatments include one fast-acting potent drug to clear parasites quickly (an artemisinin derivative) and one slower-acting partner drug to eliminate residual parasites and prevent recrudescence^72^. With these formulations, when neither active pharmaceutical ingredient (API) is SF, the artemisinin derivative is always protected by the partner API in vivo, as artemisinin is only ever present when there are high levels of the partner drug. Later in the course of infection, once the artemisinin derivative has been eliminated, the partner drug is left unprotected^73^. Protection in this context means that when both drugs are only present together at the right concentration, the probability of a resistant strain emerging is reduced by orders of magnitude, as resistance needs to develop to both drugs simultaneously, and is a key benefit of combination therapies^74^. There have been reports of both drugs in artemether-lumefantrine combination therapy containing reduced concentrations of both APIs, both separately and together, in Uganda^75^, as well as high rates of substandard combination therapies in Ghana^76^. When either of the APIs in an artemisinin-based combination are substandard, this could lead to no or reduced protection of the other drug.

For malaria, resistance typically only emerges following one or more recrudescences, and typically requires the patient to be hyperparasitaemic and/or receive a low dose^17^. If the artemisinin derivative is substandard or falsified it may have a low API or low bioavailability, both of which would increase the potential for hyperparasitaemia (Box 1B), and hence the emergence of resistance genes, which could then be selected for by the slower-acting complementary antimalarial. It will also lead to a greater risk of recrudescence, as the lower level of artemisinin derivative fails to reduce parasite numbers to a low enough level^17^ (Box 1C). When the complementary antimalarial is SF, we may again see higher parasite numbers and consequently higher rates of recrudescence and onward transmission, including of resistant parasites (Box 1C). In this case, the artemisinin-derivative could also be left unprotected (Box 1D), increasing the chance of artemisinin-resistance emerging, which could be devastating given its key role in malaria treatment and the lack of alternatives in the production pipeline. In the case that both APIs in the combination are in limited amount or not present, there is an increased risk of hyperparasitaemia due to inadequate treatment, which could then lead to an increased risk of resistance emergence if followed with treatment with a standard course, or a course in which just one of the APIs is SF (Fig. 3)^7^.

While fully artemisinin-resistant parasite strains are yet to emerge, partially resistant strains have emerged, first in Southeast Asia, and more recently in Africa^77–80^. These partially-resistant strains exhibit slower parasite clearance following commencement of artemisinin-based combination therapy. There has been speculation that the emergence of these partially resistant strains is in part due to poor quality artemisinin antimalarials, though more evidence is needed to establish this^43^. There is also evidence that use of artesunate monotherapy is associated with subsequent clonal expansion of artemisinin-resistance in Uganda^81^, which gives some indication of the danger of giving inadvertent monotherapy due to substandard partner drugs.

**Figure:**
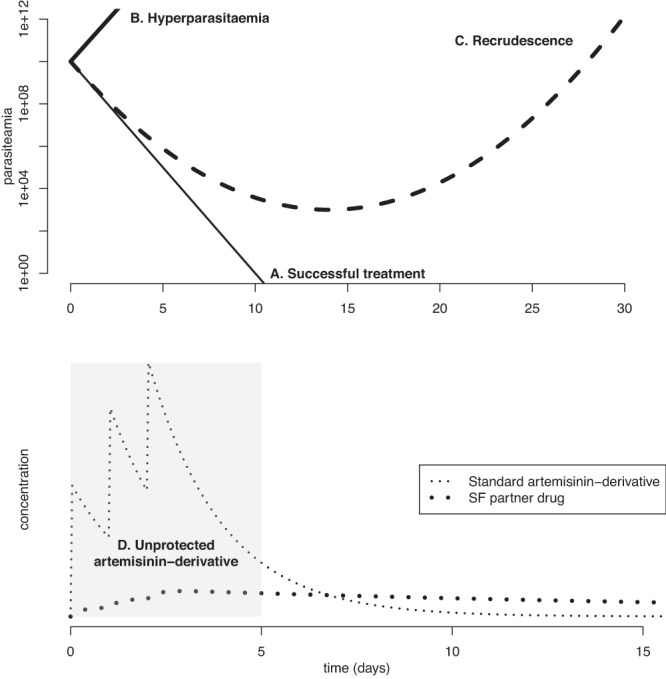
**Box Fig. 1** Substandard and falsified (SF) medicines can undermine artemisinin-combination therapies (ACTs) in several ways. **A** Under normal circumstances, the ACT at the correct dose should lead to clearance of parasites. **B** An SF artemisinin-derivative with too little active pharmaceutical ingredient (API) could lead to hyperparasitaemia. **C** If either (or both) of the APIs in the ACT are SF, there is an increased risk of recrudescence. D. If the partner drug is SF, then the artemisinin-derivative could be left unprotected due to the partner drug’s reduced concentration. Upper panel based on Fig. 1 in White et al.^17^.

## Improving our understanding

There are still many unknowns when it comes to understanding the impact of SF medicines on AMR. Improved understanding is important as, with the heterogeneous epidemiology through time and space of SF antimicrobials, their relative importance in different countries and communities as AMR drivers will vary. With multiple drivers and interventions to attempt to reduce AMR, evidence to prioritize local interventions is important to maximize impact. Solving these will require an array of new data and analytical approaches. Other causes of inappropriate dosing, unrelated to the medicine’s chemical quality, such as poor prescribing, patient adherence, and our poor understanding of the dose-response relationships for many less common pathogens, also risk AMR in similar ways to SF. If poor prescribing and adherence coincide with higher prevalence of SF medicines, their relative impact will be hard to tease apart and are likely to be synergistic.

Due to multiple overlapping drivers and the complex non-linear relationship between antibiotic consumption and the prevalence of resistance^44^, understanding this problem will also likely require the triangulation of different evidence streams^45^. This is where multiple lines of evidence from studies with different methodologies are combined to provide a more complete and convincing understanding of an issue, with the aim that the limitations and biases of each approach cancel out. In the case of SF medicines and AMR, such triangulation will likely involve ecological analysis looking at spatiotemporal correlations between SF medicines, and accounting for any lagged effects in these patterns. But it could also involve re-analyses of longitudinal studies of different doses of antimicrobials, within-host, between-host, and coupled mathematical models, in vitro studies, and pharmacokinetic-pharmacodynamic models. In the remainder of this section, we discuss these methods and the types of data that they would require in more detail.

While it would be unethical to directly observe the effects of patients taking SF medicines, it may be possible to glean information from studies which observe outcomes at different doses of antibiotics. For example, longitudinal data on the carriage of resistance genes following different doses of antibiotics, or mg/kg body weight variability, could be informative of the emergence of resistance following treatment with SF medicines. Such data could be analyzed using within-host models that explicitly model the pharmacokinetics, pathogen dynamics, and loss and acquisition of resistance^26,46,47^. A mechanistic modeling approach such as this may then enable us to extrapolate to dosing levels which are not used in clinical practice but may be present in SF medicines. Similar types of approaches may also help us to understand the likely impacts on bystander selection and combination therapy. Clearly, though, the impact of SF medicines on the emergence of resistance would depend intimately on the pathogen and antibiotic combination being considered.

Coupling the understanding gained about within-host processes with epidemiological models could help understand the population-level effects of SF medicines. An important data source to inform such models will be objective random surveys of the frequency of SF medicines, including on the % API present and the neglected aspect of bioavailability, as this will be a key determinant of the direction and strength of the effect on AMR^5,8,48^. The Medicine Quality Scientific Literature Surveyor (https://www.iddo.org/mqsurveyor/)^49^, which collates scientific articles which report the frequency of SF medicines, is one such resource, and includes only 30 random surveys of the prevalence of SF antibiotics conducted between 1992 and 2020, and many more surveys using convenience samples and other sampling approaches^4^. The World Health Organization is also currently undertaking large random surveys of the prevalence of SF medicines in several countries in sub-Saharan Africa^50^. Studies involving dissolution testing to predict the bioavailability and therapeutic efficacy of medicines are key to understanding the overall impact of SF medicines on AMR, but are currently scarce in the literature^9,48^. It is also important to undertake longitudinal studies of SF medicine frequency, % API, and bioavailability; most current studies are cross-sectional^5^. These data on SF medicines should then be complemented with data on AMR prevalence. The Global Research on AntiMicrobial resistance project (https://www.tropicalmedicine.ox.ac.uk/gram) has compiled a database on AMR prevalence that could be a key resource in this regard^11^. It will also be necessary to take a One Health perspective and consider the frequency of SF veterinary medicines and their impact on human, animal and environmental health^51,52^.

Epidemiological models could also help us understand the impact of spatio-temporal clustering of the distribution of SF medicines. For instance, certain places will likely have higher burdens of SF medicines, and their use will change through time, perhaps when an entire batch is determined to be substandard or when a covert sale of falsified medicines has recently been made^49,53,54^. Such heterogeneities can be explicitly incorporated into mathematical models. For example, mathematical models have previously been used to show how within-host heterogeneity in antibiotic concentrations could affect the emergence of resistance in cancer^55^, HIV^56^, and *M. tuberculosis*^57^, finding that such heterogeneity can both facilitate and reduce the emergence of resistance. Another important heterogeneity is the frequency of resistant strains in the community, which will have an important impact on the within-host dynamics of SF medicines^29^ and in turn on the transmission of resistant strains. Data on the frequency of resistance, and the level of resistance (i.e., the minimum inhibitory concentration), and coupled within- and between-host models could help understand this feedback across scales^16,58^. The level of resistance in a community is also likely to follow a continuous distribution, rather than being a binary trait, and models in the past have been used to understand this distribution^59^, which is likely to be relevant for the impact of SF medicines. There has been minimal laboratory work published to provide evidence to inform these discussions^12,13,60^. Further such work, including exploration of the use of hollow fiber systems^61^ may help inform discussions of the impact of SF antimicrobials on AMR in humans and other vertebrates with in vitro modeling.

Reducing the burden of SF medicines will require an international and multifaceted approach. Key will be catalyzing political will and change so that all countries have functional medicine regulatory authorities, tasking with ensuring the quality of their medicine supply. Improved risk-based post-market surveillance for SF medicines will help inform where interventions should be targeted, within the WHO Prevent, Detect and Respond strategy^4,5^. Surveillance could be improved through empowering medicine inspectors with the deployment of affordable and easy-to-use technologies that can detect both substandard and falsified medicines^62,63^. Improved regulation and monitoring of the manufacturing of medicines is also critical, including drug registration and WHO prequalification of medicines, for example^48,64^. WHO prequalification involves evaluation of certain classes of medicines according to standards of safety, efficacy, and quality, and has been associated with more than ten times lower antimalarial failure rates^6^. It is currently planned to expand prequalification to other classes of medicines^65^. A related aspect is reducing degradation during the transportation of medicines, for instance through improved supply chain logistics and storage^48,66^. A final important aspect is improving our understanding of the manufacture and trade of falsified medicines to inform and prioritize actions and interventions to reduce their occurrence. Part of this could involve innovative forensic approaches using environmental DNA and stable isotope analysis to understand the origins of falsified medicines^67,68^. Social network analysis could then help identify common trade routes and manufacturing hotspots, and hence where interventions might be most effectively targeted^69^. There have been at least two previous reviews of strategies to reduce the burden of falsified medicines^64,70^.

Substandard and falsified medicines represent a scourge on health systems through large parts of the global South, and will likely lead to large health and economic costs if we do not invest in research and systems to reduce their prevalence. This includes efforts to understand their burden, their direct impact on health outcomes, the trade-routes of falsified medicines, and what types of mechanisms could ensure the quality of antimicrobials. Solving the mystery of their impact on the burden of AMR is one critical part of this endeavor.

### Reporting summary

Further information on research design is available in the Nature Portfolio Reporting Summary linked to this article.

### Supplementary information

Supplementary Information

Reporting Summary
