# Supplementary material for: The uncertain role of substandard and falsified medicines in the emergence and spread of antimicrobial resistance
Source: Nat Commun. 2023 Oct 3;14:6153. doi: 10.1038/s41467-023-41542-w (PMC10547756; doi:10.1038/s41467-023-41542-w)
Supplement: Supplementary file 1 — Supplementary Information [file 41467_2023_41542_MOESM1_ESM.pdf]

Supplementary material for: The uncertain role of substandard and falsified medicines in the emergence and spread of antimicrobial resistance

Fig S1

Fig S2

Fig S3

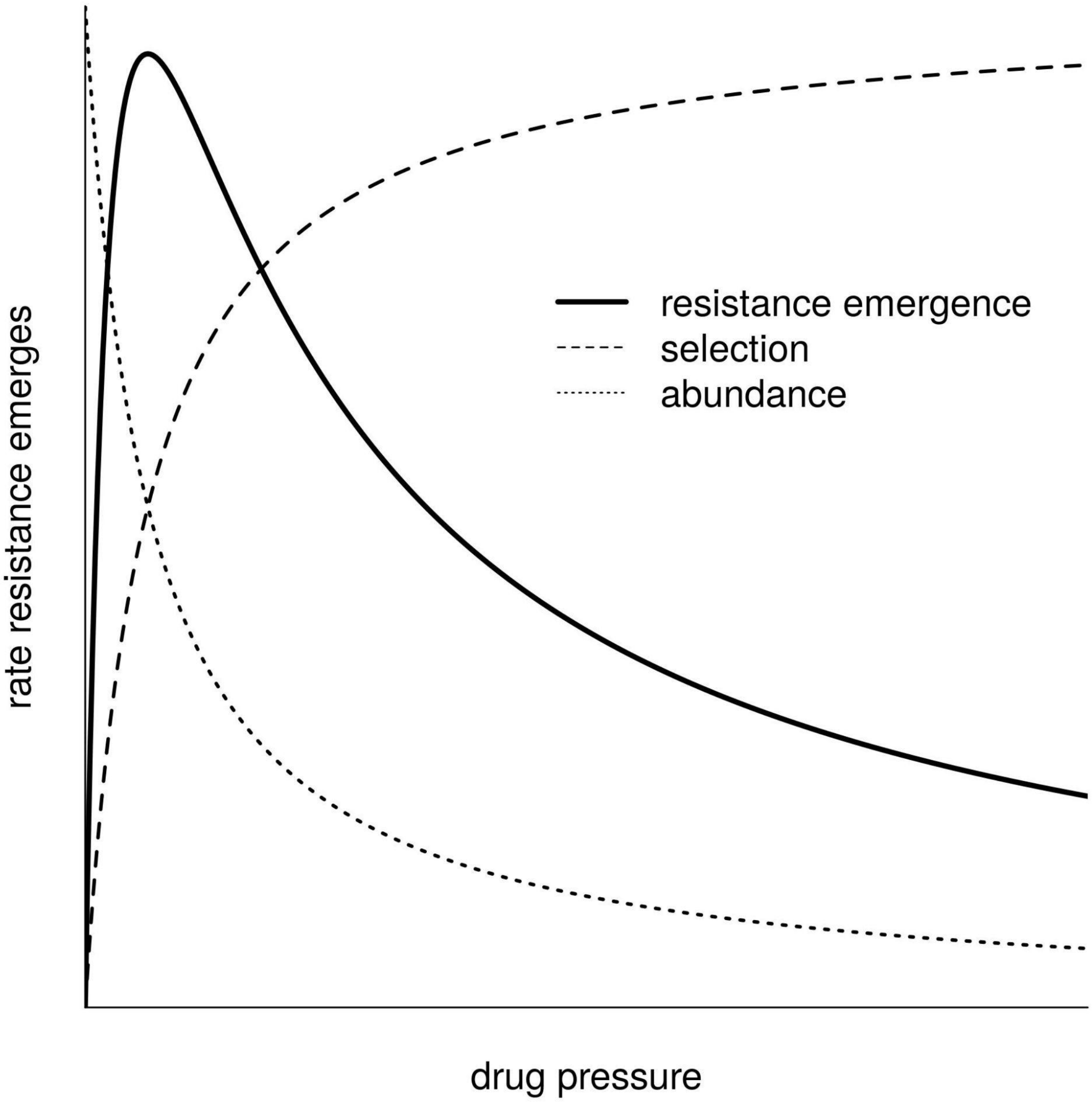

Fig S1: An example of a skewed inverted-U relationship between drug pressure and the rate of resistance emergence. If both the selective advantage of resistance and the pathogen abundance have saturating relationships with drug pressure, then the relationship between drug pressure and the rate of resistance emergence will be positively skewed, as shown here.

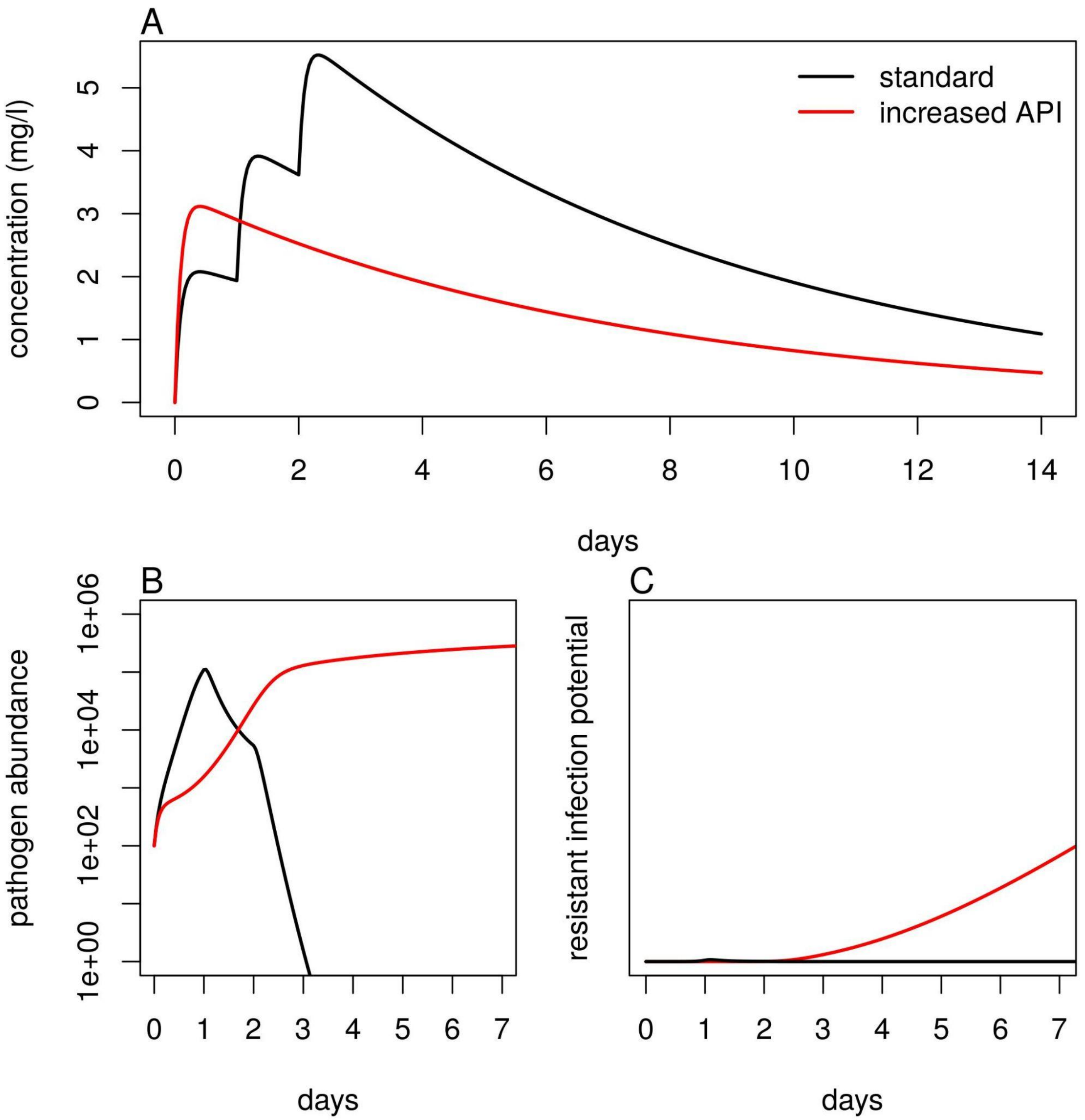

Fig S2: Medicines with increased levels of active pharmaceutical ingredients (API) can contribute to the emergence of resistance even when treatment is stopped due to increased toxicity. The black lines represent a standard regimen taken at time 0, while the red lines represent a regimen with an increased % API and stopped after the first dose. All model equations to derive these plots are as in Fig 3. A: Example concentration curves from a pharmacokinetic model with an exponential absorption and exponential elimination. The standard regimen consists of once daily treatment taken for three days - despite a lower abundance early in the infection, stopping high API treatment early fails to clear the pathogen. B: Example pathogen abundance curves. C: Example resistant infection potential curves -

stopped high API treatment leads to an increased potential for transmission of a resistant infection.

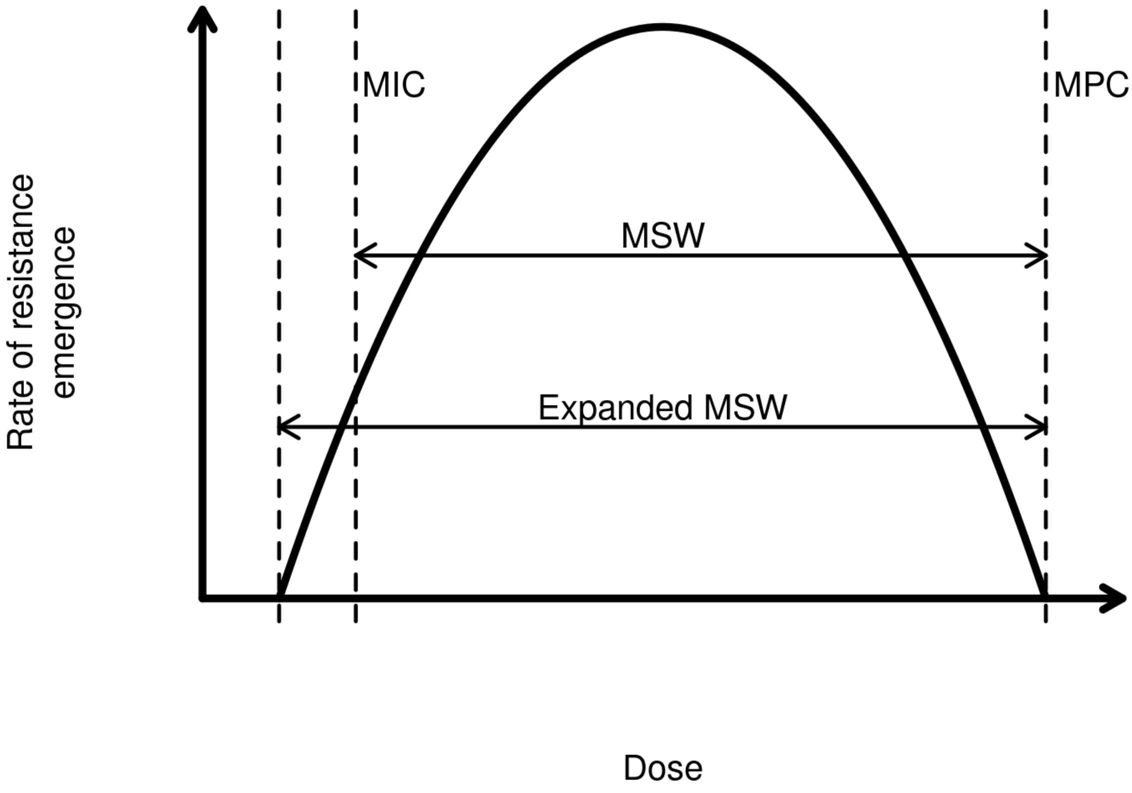

Fig. S3: The relationship between the concepts of minimum inhibitory concentration (MIC), mutant prevention concentration (MPC), and mutant selection window (MSW). The inverted-U expands these concepts by indicating that there are sub-MIC doses which can still select for resistance, and by emphasizing that some doses within the MSW lead to higher rates of resistance emergence than others.
